# Mechanical Properties and Reliability of Parametrically Designed Architected Materials Using Urethane Elastomers

**DOI:** 10.3390/polym13050842

**Published:** 2021-03-09

**Authors:** Jun Morita, Yoshihiko Ando, Satoshi Komatsu, Kazuki Matsumura, Taisuke Okazaki, Yoshihiro Asano, Masashi Nakatani, Hiroya Tanaka

**Affiliations:** 1 JSR Corporation, Yokkaichi, Mie 510-8552, Japan; Yoshihiko_Andou@jsr.co.jp (Y.A.); Satoshi_Komatsu@jsr.co.jp (S.K.); Kazuki_Matsumura@jsr.co.jp (K.M.); 2 Keio Research Institute at SFC, Keio University, Fujisawa, Kanagawa 252-0882, Japan; kirinsan@sfc.keio.ac.jp (T.O.); asanoy@sfc.keio.ac.jp (Y.A.); mn2598@sfc.keio.ac.jp (M.N.); htanaka@sfc.keio.ac.jp (H.T.)

**Keywords:** 3D printing, additive manufacturing, lattice, foam, architected material, metamaterial, elastomer, insole, reliability, Asker hardness

## Abstract

Achieving multiple physical properties from a single material through three-dimensional (3D) printing is important for manufacturing applications. In addition, industrial-level durability and reliability is necessary for realizing individualized manufacturing of devices using 3D printers. We investigated the properties of architected materials composed of ultraviolet (UV)-cured urethane elastomers for use as insoles. The durability and reliability of microlattice and metafoam architected materials were compared with those composed of various foamed materials currently used in medical insoles. The hardness of the architected materials was able to be continuously adjusted by controlling the design parameters, and the combination of the two materials was effective in controlling rebound resilience. In particular, the features of the architected materials were helpful for customizing the insole properties, such as hardness, propulsive force, and shock absorption, according to the user’s needs. Further, using elastomer as a component led to better results in fatigue testing and UV resistance compared with the plastic foam currently used for medical purposes. Specifically, polyethylene and ethylene vinyl acetate were deformed in the fatigue test, and polyurethane was mechanically deteriorated by UV rays. Therefore, these architected materials are expected to be reliable for long-term use in insoles.

## 1. Introduction

Three-dimensional (3D) printing achieves rapid shape prototyping by virtue of the direct design and manufacture of 3D shapes without the need for 2D drawings or mold fabrication [1]. Particularly in the medical field, 3D printing can be tailored to meet individual needs, e.g., for manufacturing personalized jigs and orthotics [2]. This advantage makes 3D printing preferable to the traditional process of using plaster molds in areas such as orthopedic medicine [3]. This new potential use is also expanding business models from prototyping to manufacturing, especially for “personalized manufacturing” applications [4]. However, the materials that can be used for each device are limited in 3D printing, which in turn limits the degree of freedom in designing the physical properties of the products [2,3]. Therefore, achieving multiple physical properties from a single material through 3D printing is important for expanding manufacturing applications.

In recent years, by applying 3D printing via laser sintering of metal powders [5,6], researchers have studied architected materials that exhibit unique nonlinear properties, such as structure-derived energy absorption, despite being built from a single material using a microstructured periodic lattice structure [7,8]. In addition, some researchers have attempted to apply architected material techniques to polymer materials to produce nonlinear properties [9,10]. Microstructures have been fabricated using ultraviolet (UV) light-curable elastomers, and the relationship among the 3D design parameters, deformation behavior, and mechanical properties of flexible lattice structures has been investigated [11,12,13]. Manufacturing devices using such a microlattice structure with a 3D printer can be applied to create flexible wearable materials.

However, to treat such a periodic structure as the internal structure of the device, the unit structure must be sufficiently small with respect to the actual device shape. Light and thin devices are important for medical wearable materials [3]. Therefore, the unit cell structure also needs to be small. For example, an elastomer microlattice at the micrometer scale is assigned to the shape of a wearable device (insole) having a thickness at the millimeter scale when considering the external shape and local hardness of the device [14].

It remains unclear, however, whether the durability and reliability of such microlattice structures are sufficient, particularly in comparison with those of materials currently used in medical insoles. Achieving industrial-level durability and reliability is necessary for individualized manufacturing of these devices using 3D printers.

Traditionally, foamed plastic polymer materials such as polyethylene (PE) foam, ethylene vinyl acetate (EVA) foam, and polyurethane (PU) foam have been used in medical insoles [15,16,17]. In particular, polyurethane stands out as a material with excellent hardness, impact absorption, and rebound resilience for custom-made insoles for medical and sports applications [16,17]. On the other hand, the density and hardness of polyurethane foam are trade-offs [18]. Notably, the properties of these foam materials depend on whether the cell bubbles are continuous or closed bubbles [19]. For medical insoles, it is important to consider the many ways in which they might be used [20]; continuous-bubble foam responds quickly to the loading and unloading process but is softer. On the other hand, closed-bubble foam is harder and exhibits viscoelastic behavior, but these properties gradually change with repeated loading and unloading [21]. Furthermore, the degradation of materials due to heat, UV light, and moisture must also be considered [22,23]. Elastomers have been used in medical applications and are known to exhibit excellent physical strength, toughness, and durability [24]. If flexible physical properties in a non-foam material can be achieved by an architected material made of an elastomer, it is likely that both durability and other physical properties can also be simultaneously achieved as a material for insoles. However, no series of studies comparing the physical properties of such architected materials with those of existing foam materials has yet been conducted.

In this study, we investigate the properties of architected materials made from UV-cured urethane elastomers. To consider the practicality of such materials for insoles, the durability and reliability are compared with those of foam materials used in existing medical insoles.

## 2. Materials and Methods

### 2.1. Materials

#### 2.1.1. Architected Material 1 (Microlattice)

**Figure 1:**
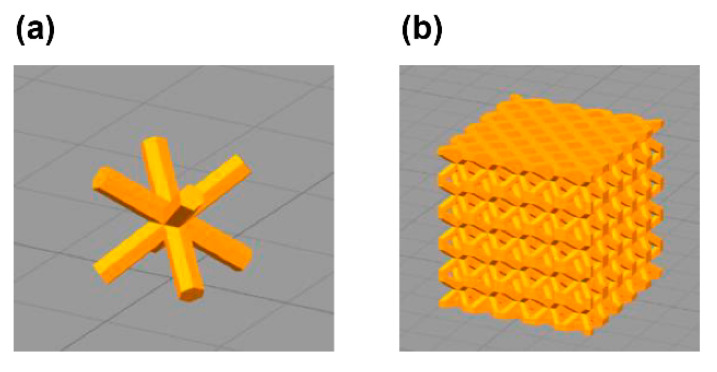
3D structures of architected materials designed in OpenSCAD [25]: (**a**) unit cell structure; (**b**) 5 × 5 × 5 units lattice cube structure.

To investigate the characteristics of the microlattice, each microlattice was prepared with an adjusted design parameter. OpenSCAD [25] can generate 3D structures via scripting, and the structures can be designed parametrically. In this study, columns were placed on the unit cell based on the body-centered cubic structure and the pattern shown in Figure 1a. The size of the unit lattice was 4 × 4 × 4 mm^3^, and a 20 × 20 × 20 mm^3^ cube structure was designed in which the unit cell was periodically arranged in 5 × 5 × 5 units, as shown in Figure 1b. In the architected material, the diameter of each column of the unit lattice was changed from 0.80 to 1.52 mm. To make the contact area uniform, each cube had a bottom and top plate, each 0.7 mm thick. To maintain ventilation, square holes (1.7 × 1.7 mm^2^) were positioned periodically on each side of each of the bottom and top plates. UV-cured urethane elastomer EPU41 (Carbon Inc., Redwood city, CA, USA) was used for UV modeling using an L1 photo-curing 3D printer (Carbon Inc., Redwood city, CA, USA). Finally, the structures made of architected materials were created via heat treatment at 120 °C for 8 h (hours). In particular, the physical properties and UV resistance of the samples with column diameters of 0.80 (ML-1), 1.20 (ML-2), and 1.52 mm (ML-3) were compared with those of the plastic foamed materials discussed in Section 2.1.3. The characteristics and photos of these microlattices are shown in Table A2 and Figure A2 and Figure A3. Three different sample sizes were fabricated: 20 × 20 × 20 mm^3^, 110 × 60 × 5 mm^3^, and 50 × 20 × 5 mm^3^.

#### 2.1.2. Architected Material 2 (Metafoam)

**Figure 2:**
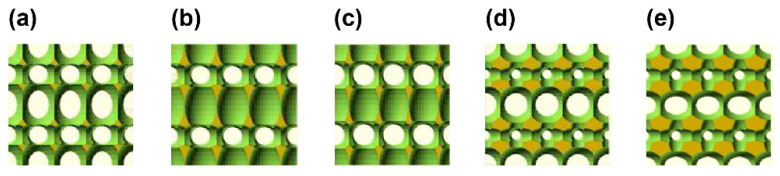
3D structures of metafoams designed in OpenSCAD [25]; (**a**) MF-1; (**b**); MF-2; (**c**) MF-3; (**d**) MF-4; (**e**) MF-5.

**Figure 3:**
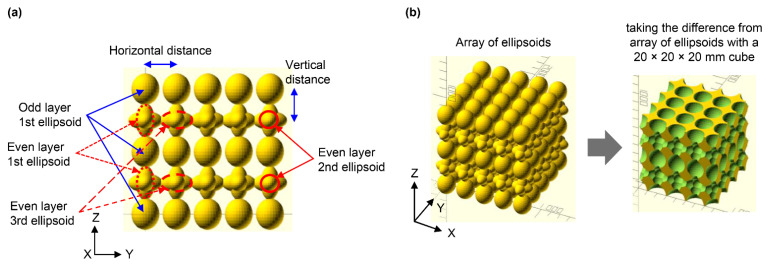
3D structures of architected materials designed in OpenSCAD [25]: (**a**) placement of ellipsoids; (**b**) metafoam structure obtained through the difference between the ellipsoidal array and 20 × 20 × 20 mm^3^ cube.

Metafoam is an architected material with artificially arranged bubbles. To investigate the characteristics of this material, five metafoam structures (MF-1, MF-2, MF-3, MF-4, MF-5) with adjusted design parameters, shown in Figure 2, were created using OpenSCAD and were fabricated. The metafoam was created by difference set using a cube with bubble sizes designed parametrically. First, four types of ellipsoids were placed at fixed intervals (5 mm) in the horizontal and vertical directions, giving different ellipsoid sized for odd and even layers, as shown in Figure 3a. In odd layers, one type of ellipsoid was placed wherein size was represented by rx, ry, and rz. In even layers, three types of ellipsoids were placed. The first, second, and third ellipsoid sizes were represented by lx, ly; lz, sx, and sy; and sz, tx, ty, and tz, respectively. The x, y, and z symbols represented the *x*-axis, *y*-axis, and *z*-axis directions, respectively. The detailed design values for each size parameter are shown in Table A1. Finally, we designed the metafoam by subtracting an ellipsoid array from a cube having a size of 20 × 20 × 20 mm^3^, as shown in Figure 3b. UV-cured urethane elastomer EPU41 was used for UV modeling using the aforementioned L1 3D printer. Finally, the structures made of architected materials were created via heat treatment at 120 °C for 8 h. The characteristics of these metafoams are shown in Table A3 and in Figure A3.

#### 2.1.3. Polymer Foam

**Figure A1:**
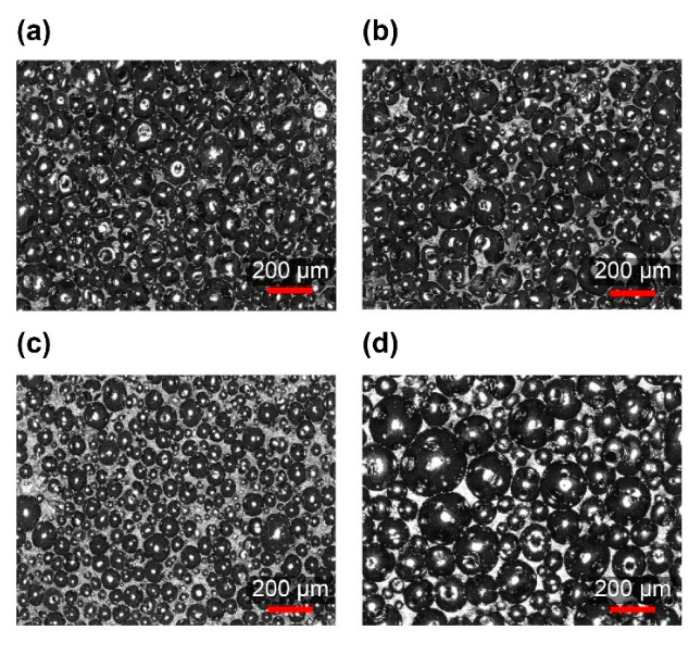
Cross-section images of each PU foam were observed by laser microscope. (**a**) PUfoam-1; (**b**) PUfoam-3; (**c**) PUfoam-5; (**d**) PUfoam-7.

**Figure A2:**
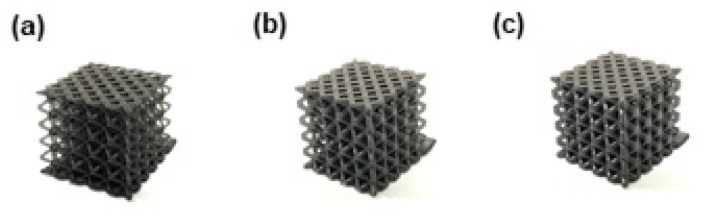
Photographs of microlattices obtained by the method discussed in Section 2.1.1. (**a**) ML-1; (**b**) ML-2; (**c**) ML-3.

**Figure A3:**
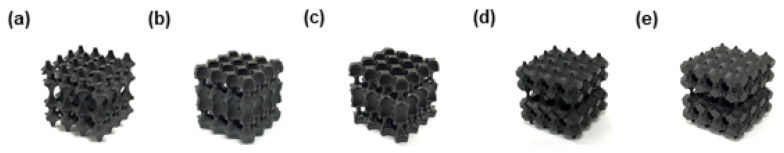
Photographs of metafoams obtained by the method discussed in Section 2.1.2. (**a**) MF-1; (**b**) MF-2; (**c**) MF-3; (**d**) MF-4; (**e**) MF-5.

To compare the properties of architected materials and plastic foam used in existing medical insoles, foam materials of various densities (EVAfoam-1, EVAfoam-2, EVAfoam-3, PEfoam, and PUfoams-1 to -7) were obtained. Their apparent densities and the Asker C hardness of a 20 × 20 × 20 mm^3^ cube were measured, as shown in Table A4. For EVAfoam-1, EVA foam with a hardness of 70 was purchased from Benkyodo Co., Nagoya, Japan; the apparent density of a cube of dimensions 20 × 20 × 20 mm^3^ was 0.28 g/cm^3^. For EVAfoam-2, EVA foam P-E Lite (A-20) was purchased from INOAC Corp., Nagoya, Japan; the apparent density of a cube of dimensions 20 × 20 × 20 mm^3^ was 0.18 g/cm^3^. For EVAfoam-3, EVA foam nora^®^ Lunairmed was purchased from Nora Systems, Inc., Salem, NH, USA; the apparent density of a cube of dimensions 20 × 20 × 20 mm^3^ was 0.09 g/cm^3^. For PEfoam, PE foam AZOTE^®^ (LD-45) was purchased from INOAC Corp., Nagoya, Japan; the apparent density of a cube of dimensions 20 × 20 × 20 mm^3^ was 0.05 g/cm^3^. For PUfoams-1 to -6, PU foam PORON^®^ foams L-24, L-32, H-24, H-32, H-48, and HH-48 were purchased from ROGERS INOAC Corp., Nagoya Japan; for these foams, the apparent densities of a cube of dimensions 20 × 20 × 20 mm^3^ ranged from 0.24 to 0.52 g/cm^3^. For PUfoam-7, PU foam X2 SOFT/MAROON was purchased from Henry Schein, Inc., Melville, NY, USA; the apparent density of a cube of dimensions 20 × 20 × 20 mm^3^ was 0.05 g/cm^3^. Laser microscopic images of each PU foam’s cross-section are shown in Figure A1.

### 2.2. Methods

#### 2.2.1. Apparent Density and Porosity

This evaluation was performed to measure the apparent density and porosity of each material. Each cube sample designed with dimensions of 20 × 20 × 20 mm^3^ was weighed, and the actual dimensions were measured using a Vernier caliper. The apparent density was calculated by taking the ratio of the weight to the volume calculated from the actual dimensions. The porosity values of the architected materials were calculated by taking the ratio of the bulk density of EPU41 to the apparent density of the architected material.

#### 2.2.2. Hardness

This evaluation was performed to measure the hardness of each sample. The hardness (Asker C) was measured using a rubber hardness tester (C1L) equipped with a constant pressure loader (CL-150) manufactured by Polymer Instrument Co., Kyoto, Japan. The hardness was considered to be the peak value for a sample of dimensions 20 × 20 × 20 mm^3^ pressed with a constant load of 9.8 N. The average value of the three measurements was taken as the hardness.

#### 2.2.3. Rebound Resilience

This evaluation was performed to measure the rebound resilience of each sample. A shovel-type repulsive elasticity tester (RT-90) manufactured by Polymer Instrument Co., Kyoto, Japan was used to strike a sample of dimensions 20 × 20 × 20 mm^3^ with a pendulum six times, and the average of the three measurements from the fourth strike onward was considered as the rebound resilience.

#### 2.2.4. Frictional Force

This evaluation was performed to measure the grip performance of each sample. The frictional force was measured using a Tribo Station (Type:32), a surface property measurement instrument manufactured by Shinto Scientific Co., Ltd., Tokyo, Japan. A flat indenter with dimensions 30 × 30 mm^2^, laminated with a non-woven waste cloth, was pressed against a specimen of dimensions 110 × 60 × 5 mm^3^ at a load of 200 g. The dynamic frictional coefficient was evaluated when the specimen was scanned at a moving speed of 500 mm/min and a reciprocating distance of 40 mm.

#### 2.2.5. Hysteresis Loss

This evaluation was performed to measure the energy loss in one compression–release cycle of each sample. The hysteresis loss was measured using a compression tester (Instron S5976) manufactured by Illinois Tool Works Inc., Glenview, IL, USA. A sample of dimensions 20 × 20 × 20 mm^3^ was compressed at a rate of 10 mm/min to a displacement of 50% of the thickness of the sample and then released at a rate of 10 mm/min. The ratio of the area surrounded by compression and release to the area surrounded by compression was recorded as hysteresis loss.

#### 2.2.6. Ultraviolet Light Resistance

This evaluation was performed to consider the risk of degradation when each sample was exposed to UV light. Specimens of dimensions 50 × 20 × 5 mm^3^ were irradiated for 48 h at 63 °C in a chamber using a Suga Test Instruments Co. Ltd., Tokyo, Japan, Testing Machine ultraviolet carbon arc fade meter (U48). Changes in appearance after irradiation were visually observed, as was the presence of cracks after the specimen was bent by hand. In addition, a tensile tester (Instron S5976) was used to pull the two samples at a rate of 500 mm/min before and after irradiation.

#### 2.2.7. Compression Fatigue Test

This evaluation was performed to consider the dent performance when each sample was repeatedly subjected to loading–unloading. A specimen of dimensions 20 × 20 × 20 mm^3^ was used to perform a 10,000-cycle compression fatigue test at a frequency of 1.25 Hz with an average load of 0.16 kN and an amplitude of 0.16 kN using a 5-kN fatigue testing machine manufactured by Tokohoki Co., Tokyo, Japan. After the fatigue test, the sample thickness was measured after standing at room temperature for 0.5 h. The ratio of the thickness after the test to the thickness before the test was calculated.

## 3. Results and Discussion

### 3.1. Microlattice Compared with Plastic Foam

This section compares the physical properties and UV resistance of the microlattices and plastic foams used in existing medical insoles (EVAfoam-1, EVAfoam-2, EVAfoam-3, PEfoam, and PUfoam-7).

**Figure 4:**
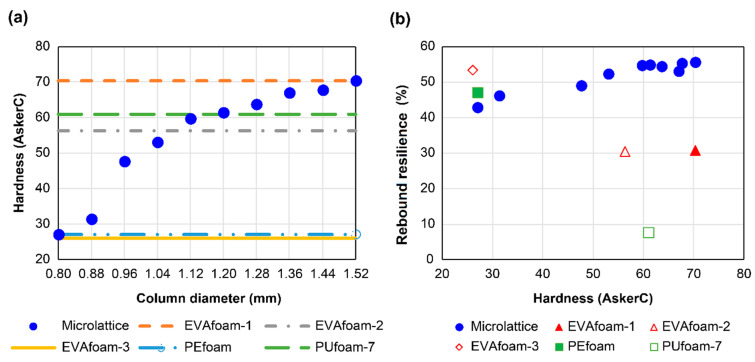
Comparative results for mechanical properties: (**a**) relationship between diameter of pillars in the unit cell and hardness (Asker C) for the microlattice structures; (**b**) relationship between hardness (Asker C) and rebound resilience.

The hardness (Asker C) of the lattice cube after the column diameter of the unit lattice was changed from 0.80 to 1.52 mm and that of the foams used in existing medical insoles is shown in Figure 4a. For the microlattice, the hardness increased as the diameter of the pillars increased. Furthermore, adjusting the column diameter of the microlattice enabled it to achieve the same hardness value as that of each foam material. The hardness of foam materials is attributed to the type of plastic and the density of the foam. In contrast, it was confirmed that the microlattice was composed of a single material and that the hardness can be freely controlled only by the column diameter.

The relationship between the hardness and rebound resilience of each microlattice and foam material is shown in Figure 4b. It was found that the microlattice structures had high rebound resilience for a wide hardness range. This behavior is attributed to the characteristics of the elastomer material and the low energy dissipation of the structured material when a body-centered cubic lattice was used as the unit lattice. In particular, such lattices exhibited a load–displacement curve in which the structure did not buckle [14].

**Figure 5:**
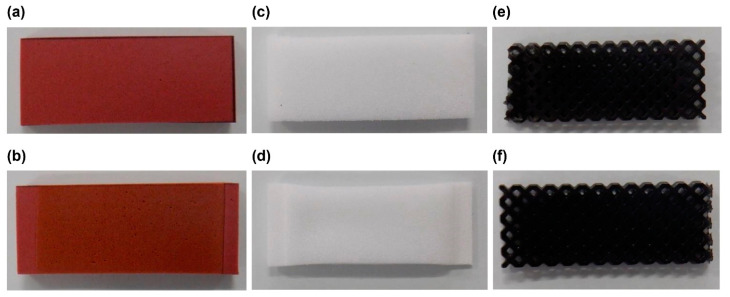
Appearance of samples before and after carbon arc testing: (**a**) PUfoam-7 before carbon arc testing; (**b**) PUfoam-7 after carbon arc testing (discoloration occurred); (**c**) PEfoam before carbon arc testing; (**d**) PEfoam after carbon arc testing (shrinkage occurred); (**e**) microlattice before carbon arc testing; (**f**) microlattice after carbon arc testing (did not occur).

**Figure 6:**
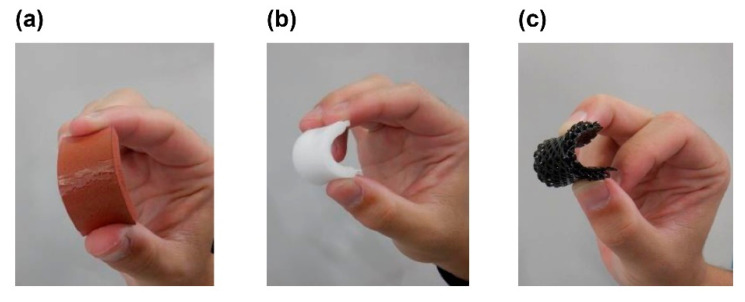
Appearance of samples after bending: (**a**) PUfoam-7 (cracking occurred); (**b**) PEfoam; (**c**) microlattice sample.

**Figure 7:**
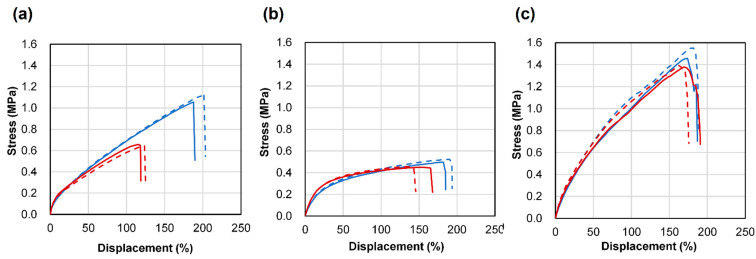
Stress–strain curve obtained by tensile test of each sample before (blue line) and after (red line) carbon arc testing. The solid (dashed) line indicates first (second) sample. (**a**) PUfoam-7; (**b**) PEfoam; (**c**) microlattice sample.

The appearance changes related to UV resistance are shown in Figure 5 and Figure 6. EVAfoams-1 to -3 shrank significantly after irradiation and thus are not shown. After 48 h of carbon arc testing, PUfoam-7 became discolored, and the PEfoam shrank, as shown in Figure 5. In addition, when the samples were bent after the test, cracks occurred in PUfoam-7, as shown in Figure 6. In contrast, the microlattice samples showed no abnormality in appearance or bending after the test. These appearance changes are considered to be related to the deterioration of the material. Previous studies have reported that when urethane foam is exposed to UV light, it undergoes photodegradation and photooxidation, as confirmed by color changes and Fourier transform infrared (FTIR) spectroscopy results [26]. In this study, the deterioration of the material was determined by evaluating the mechanical properties by performing tensile tests on the sample before and after irradiation. The results of the stress–displacement curve of each sample are shown in Figure 7. The stress–displacement curves of PUfoam-7 and PEfoam clearly changed before and after irradiation. Specifically, the elongation at break after irradiation decreased. In the microlattice, no change was observed in the stress–displacement curve before and after irradiation. It is presumed that the changes in the appearance and mechanical properties of PUfoam-7 after the carbon arc test were caused by photodecomposition and photooxidation. The change in mechanical properties in the PEfoam could be related to the non-uniform shrinkage of the sample, as shown in Figure 5d. From these results, it is clarified that the microlattices have excellent UV resistance compared with the foam materials used in existing medical insoles.

The characteristics of each material, including the coefficient of friction, in addition to the aforementioned physical properties and UV resistance, are summarized in Table 1. The Asker C hardness of the architected material, which comprises a single elastomer, could be freely changed, and the design could be aimed at the hardness of various polymer foams. Moreover, when the hardness varied from 27 to 70, the rebound resilience changed from 43% to 56%. The hardness and rebound resilience are proportional, and high rebound resilience can be maintained even at high hardness against foam materials. In addition, the coefficient of friction of the microlattices showed a constant trend against hardness, which was higher than that of foam materials used in existing medical insoles. This result indicates that the microlattices had high grip performance. Furthermore, the carbon arc test confirmed that, compared with currently used foam materials, the architected materials were more reliable in the UV environment. Microlattice structures composed of UV-cured urethane elastomers exhibited high rebound resilience and grip, and the hardness was easily tuned by adjusting the pillar diameter of the unit cell. The architected materials were also shown to be more UV-resistant than existing foam materials, suggesting that they can be air-dried outdoors after washing with water, with no substantial degradation.

### 3.2. Metafoam Structure Compared with Microlattice and Urethane Foam

This section compares the physical properties of metafoams and microlattices, which are different architected materials made from UV-cured urethane elastomer. In addition, to focus on the structural differences, urethane foams used in existing medical insoles are also compared.

**Figure 8:**
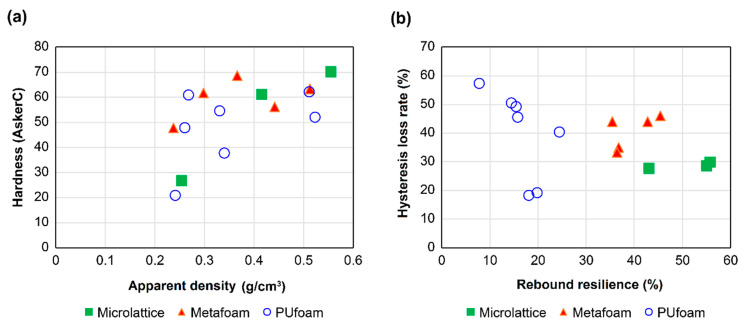
Comparative results of mechanical properties: (**a**) relationship between apparent density and hardness for microlattice structure (filled green square), metafoam structure (filled red triangle), and urethane foam (open blue circle); (**b**) relationship between rebound resilience and hysteresis loss rate for microlattice structure (filled green square), metafoam structure (filled red triangle), and urethane foam (open blue circle).

The relationship between the hardness and apparent density of each material is shown in Figure 8a. By comparing metafoams and microlattices, it was found that the relation between the hardness and apparent density was different even though they had almost the same apparent density. The hardness of the microlattices decreased as the apparent density decreased, although that of metafoams remained high even at a low apparent density. Although they are composed of the same material, the hardness can be controlled by the design pattern. In addition, these two architected materials covered the area of apparent density and hardness of urethane foams. Conventionally, these two factors in foam materials are considered to be trade-offs, controlled by the foaming ratio and the state of bubble connection [18]. However, metafoam with artificially arranged bubbles showed different results from the conventional tendency.

Figure 8b shows a plot of the rebound resilience and hysteresis loss rate of each material. The hysteresis loss rate was measured for only one cycle. Previous studies on the compression response of open-cell foams have discussed the hysteresis loss rates measured multiple times [27]. Although a cross-section of the urethane foams used for this comparison is shown in Figure A1, it was unclear whether the structure was open-cell or closed-cell. In the case of a closed-cell structure, the closed cells can be destroyed by multiple measurements; therefore, the hysteresis loss rate was measured from only one cycle. Although the metafoams and microlattices had almost the same apparent density, as shown in Figure 8a, the former had lower rebound resilience and a higher hysteresis loss rate than the latter. Furthermore, most of the urethane foams had lower rebound resilience and a higher hysteresis loss rate than those in the architected materials. A microlatice is a structure with high uniformity and low energy dissipation, as described in Section 3.1. In contrast, metafoam is a structure in which the space is non-uniform, which causes differences in rebound resilience and the hysteresis loss rate. However, metafoam has higher uniformity than urethane foams because the bubbles are artificially arranged in a regular pattern. This design is expected to provide higher rebound resilience and hysteresis loss rates than those in urethane foams used in existing medical insole.

Table 2 shows the results of the hardness, rebound resilience, and hysteresis loss tests for microlattices and for each of the metafoams and urethane foam materials.

The physical properties were changed by altering the design pattern of the architected materials composed of UV-curable urethane elastomer. The metafoam maintained high hardness even when the apparent density was low. It is suggested that metafoam structures also expand the possibilities of controlling the physical properties of insole materials.

### 3.3. Durability of Architected Materials and Plastic Foam

**Figure 9:**
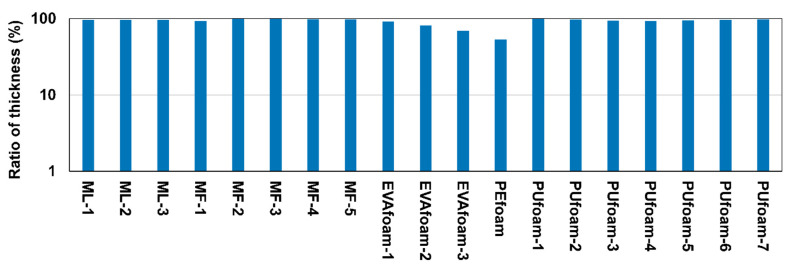
Ratio between sample thickness after a 10,000-cycle compression fatigue test and thickness before the test.

This section discusses the durability of architected materials and plastic polymer foams used in existing medical insoles to evaluate their practicality as insole materials. The ratio of sample thickness after a 10,000-cycle compression fatigue test and the thickness before the test is shown in Figure 9. The higher the ratio of the sample thickness, the smaller the permanent strain of the sample, which indicates higher durability. Although a difference was noted in the physical properties of the microlattices and metafoams, as described in Section 3.2, the ratio of thickness after the compression fatigue test was almost the same. Further, the ratio of thickness of these architected materials was higher than that of EVAfoam and PEfoam and almost the same as that of PUfoam. In light of these results, architected materials such as microlattice and metafoam are expected to be reliable in long-term use compared to EVAfoam and PEfoam.

### 3.4. Practicality as Insole Material

This section discusses the materials’ practicality as an insole material according to the aforementioned results. The physical properties, durability, and reliability of each material compared in this study are summarized in Table 3. The hardness of the architected materials can be adjusted continuously by controlling the design parameters, although this is not possible in the foamed materials. In addition, the rebound resilience can be controlled by combining microlattices and metafoams. These architected materials’ features are helpful for customizing the insole properties, such as hardness, propulsive force, and shock absorption, according to the user’s needs. In addition, the architected materials composed of UV-cured urethane elastomers exhibited higher coefficients of friction than those in the foamed materials, which resulted in high grip performance. Further, the architected materials have sufficiently high durability and reliability, which were lacking in the foamed materials. The insoles made with the architected materials are more suitable for long-term use than the existing medical insoles made by foamed materials. Therefore, the architected materials are considered to be practical as insole materials.

## 4. Conclusions

The durability and reliability of various foam materials currently used in medical insoles were compared with those of architected materials. It was shown that architected materials made of UV-cured urethane elastomers exhibited high resilience and grip, and that the hardness could easily be tuned by adjusting the pillar diameter of the unit cell. Furthermore, the body-centered microlattice exhibited higher rebound resilience and lower hysteresis loss than the metafoam structure over the same density range. Therefore, the microlattice structure with low energy loss during walking is expected to provide high propulsive force, whereas the metafoam structure is expected to absorb the shock during walking. Clearly, the design space for rebound resilience and hysteresis loss characteristics can be parametrically adjusted by altering the design patterns of lattice and foam structures. In addition, using elastomer as a component in the architected materials yielded better fatigue test results and UV resistance compared with the plastic foam currently used for medical purposes. PEfoam and EVAfoam were deformed in the fatigue test, and PUfoam was mechanically deteriorated by UV rays. Therefore, the architected materials are more reliable for long-term use in insoles.

## Figures and Tables

**Table 1:** Physical properties and UV reliability of microlattices and foam materials used in existing medical insoles.

Material	Hardness (Asker C)	Rebound Resilience (%)	Coefficient of Friction	Carbon Arc Test (48 h)
EPU41-Bulk	85 (0.6)	53 (0.6)	1.04	OK
ML-1	27 (1.0)	43 (0.6)	1.04	OK
ML-2	61 (1.2)	55 (0.6)	1.04	OK
ML-3	70 (0.6)	56 (0.6)	1.04	OK
EVAfoam-1	71 (1.5)	31 (1.0)	0.56	Shrinking
EVAfoam-2	57 (1.5)	31 (0.6)	0.71	Shrinking
EVAfoam-3	27 (0.6)	53 (0.6)	1.02	Shrinking
PEfoam	27 (0.6)	47 (0.6)	0.73	Shrinking
PUfoam-7	61 (0.6)	8 (0.6)	0.23	Discoloring/Cracking

The hardness and rebound resilience indicate the average value of the three measurements, and the numbers in parentheses indicate the standard deviation.

**Table 2:** Physical properties of architected materials built from EPU41 and urethane foams used in existing medical insoles.

Material	Apparent Density (g/cm^3^)	Hardness (Asker C)	Rebound Resilience (%)	Hysteresis Loss Rate (%)
EPU41-Bulk	1.00	85 (0.6)	53 (0.6)	33
ML-1	0.25	27 (1.0)	43 (0.6)	28
ML-2	0.41	61 (1.2)	55 (0.6)	29
ML-3	0.55	70 (0.6)	56 (0.6)	30
MF-1	0.24	48 (1.5)	35 (0.6)	44
MF-2	0.37	69 (0.6)	45 (0.6)	46
MF-3	0.30	62 (1.7)	43 (0.6)	44
MF-4	0.44	56 (2.1)	37 (0.6)	35
MF-5	0.51	64 (1.7)	36 (0.6)	33
PUfoam-1	0.24	21 (0.6)	18 (1.0)	19
PUfoam-2	0.34	38 (0.6)	20 (0.6)	19
PUfoam-3	0.26	48 (1.0)	14 (0.6)	51
PUfoam-4	0.33	55 (0.6)	15 (0.6)	50
PUfoam-5	0.52	52 (0.6)	24 (0.6)	41
PUfoam-6	0.51	62 (0.6)	16 (0.6)	46
PUfoam-7	0.27	61 (0.6)	8 (0.6)	57

The hardness and rebound resilience indicate the average value of the three measurements, and the numbers in parentheses indicate the standard deviation.

**Table 3:** Practicality of architected materials and commercial foams as an insole material.

Material	Hardness	Rebound Resilience	Coefficient of Friction	UV Resistance	Durability
ML	Controllable	High	High	Good	Good
MF	Controllable	Middle	-	-	Good
EVAfoam	Not adjustable	Middle–high	Middle	Poor	Poor
PEfoam	Not adjustable	High	Middle	Poor	Poor
PUfoam	Not adjustable	Low	Low	Poor	Good

## Data Availability

This study did not report any data.

## Appendix A. Metafoam Design Parameters

Table A1 shows the size parameters of each ellipsoid for designing the five types of metafoams (MF-1 to MF-5) shown in Figure 2. The size of the ellipsoids in the odd layers was defined by rx, ry, and rz. The size of the first ellipsoids in even layers was defined by lx, ly, and lz. The size of the second ellipsoids in the even layer was defined by sx, sy, and sz. The size of the third ellipsoids in the even layer was defined by tx, ty, and tz. The x, y, and z symbols represented the *x*-axis, *y*-axis, and *z*-axis directions, respectively. The unit shown in Table A1 is millimeter (mm).

**Table A1:** Size parameters of each ellipsoid that constitute the 20 × 20 × 20 mm^3^ metafoam.

Component	Size Parameter	(a) MF-1	(b) MF-2	(c) MF-3	(d) MF-4	(e) MF-5
Odd Layer ellipsoid	rx	6.0	5.0	5.0	6.3	6.3
Odd Layer ellipsoid	ry	6.0	5.0	5.0	6.3	6.3
Odd Layer ellipsoid	rz	8.3	8.3	8.3	6.3	5.0
Even Layer 1st ellipsoid	lx	4.0	4.0	4.8	2.5	2.5
Even Layer 1st ellipsoid	ly	4.0	4.0	4.8	2.5	2.5
Even Layer 1st ellipsoid	lz	7.3	7.3	7.3	6.3	6.3
Even Layer 2nd ellipsoid	sx	7.3	7.3	7.3	6.3	6.3
Even Layer 2nd ellipsoid	sy	4.0	4.0	4.8	2.5	2.5
Even Layer 2nd ellipsoid	sz	4.0	4.0	4.8	2.5	2.5
Even Layer 3rd ellipsoid	tx	4.0	4.0	4.8	2.5	2.5
Even Layer 3rd ellipsoid	ty	7.3	7.3	7.3	6.3	6.3
Even Layer 3rd ellipsoid	tz	4.0	4.0	4.8	2.5	2.5

## Appendix B. Cross-Section Image of PU Foam Sample

Cross-section images of each PU foam sample are shown in Figure A1. These were captured using a laser microscope (VH-X200) manufactured by KEYENCE Co., Tokyo, Japan. Bubbles with a maximum cross-sectional dimension of 20 to 200 μm were observed in each PU foam. (a) PUfoam-1 and (b) PUfoam-3 exhibited similar bubble size. (c) PUfoam-5 exhibited smaller bubbles, and (d) PUfoam-7 exhibited larger bubbles than those of (a) PUfoam-1.

## Appendix C. Photograph of Microlattice and Metafoam

Figure A2 and Figure A3 show photographs of the architected materials obtained by the method discussed in Section 2.1.

## Appendix D. Characteristics Table

Table A2, Table A3 and Table A4 show the characteristics of individual 20 × 20 × 20 mm^3^ materials, including those of the microlattices mentioned in Section 2.1.1, those of the metafoams mentioned in Section 2.1.2, and those of foamed materials mentioned in Section 2.1.3, respectively.

**Table A2:** Characteristics of individual 20 × 20 × 20 mm^3^ microlattices.

Characteristic	(a) ML-1	(b) ML-2	(c) ML-3
Weight (g)	2.02	3.31	4.43
Apparent Density (g/cm^3^)	0.25	0.41	0.55
Porosity (%)	74.8	58.7	44.9

**Table A3:** Characteristics of individual 20 × 20 × 20 mm^3^ metafoams.

Characteristic	(a) MF-1	(b) MF-2	(c) MF-3	(d) MF-4	(e) MF-5
Weight (g)	1.89	2.92	2.38	3.53	4.10
Apparent Density (g/cm^3^)	0.24	0.37	0.30	0.44	0.51
Porosity (%)	76.4	63.6	70.4	56.0	49.0

**Table A4:** Characteristics of individual 20 × 20 × 20 mm^3^ foam materials.

Characteristic	EVA Foam-1	EVA Foam-2	EVA Foam-3	PE Foam	PU Foam-1	PU Foam-2	PU Foam-3	PU foam-4	PU Foam-5	PU Foam-6	PU Foam-7
Weight (g)	2.21	1.42	0.75	0.39	1.92	2.71	2.07	2.63	4.18	4.08	2.13
Apparent Density (g/cm3)	0.28	0.18	0.09	0.05	0.24	0.34	0.26	0.33	0.52	0.51	0.27
Hardness (Asker C)	70	56	26	27	21	38	48	55	52	62	61
